# Notes on Psychoanalysis

**Published:** 1915-11

**Authors:** Hansell Crenshaw

**Affiliations:** Director Oakwood Sanitarium and Neurologist to Grady Hospital, Atlanta, Ga.


### NOTES ON PSYCHOANALYSIS.

By Hansell Crenshaw, M. D., Director Oakwood Sanitarium and Neurologist to Grady Hospital, Atlanta, Ga.

The important place which psychoanalysis is taking among methods of treating nervous and mental diseases, seems to justify the following effort at discussing some leading questions concerning it in an unpretentious way.

Briefly, psychoanalysis is a method of aleviating certain nervous and mental disorders of psychic origin by discovering hidden mental conflicts on the part of the patient and overcoming them by readjustment of ideas. For instance, one young women suffered from an incontrollable fear of red; and psychoanalysis revealed that the sight of blood under trying circumstances in early childhood had left in her mind this conflict between the perception of red and her emotions. Psychoanalysis is based upon the theory that hysteria, phobias, obsessions, incontrollable impulses, and certain insanities are simply mental conflicts due to the repression of inimicable ideas and impulses. Such represson is largely a defense mechanism on the part of the mind, but also partly compensatory. It is defensive, for example, when in our effort to protect the mind against a painful memory we finally succeed in apparently crowding the painful experience out of consciousness—succeed in forgetting it and all the circumstance associated with it. The blanks in the memories of hysteria patients are familiar to every student of the disease. Another defense mechanism is the delusion of death; a patient whose memories are unendurable thinks of death as an avenue of escape till he finally imagines that he really is dead.

Repression may become compensatory when a successful business man, for instance, fails in business and in ability, but represses the consciousness of this failure and begins to substitute the idea that he is an importanc personage or the possessor of a considerable fortune.

But how is psychoanalysis supposed to relieve these conflicts, these psychic repressions? The principle is that a discovery by an understanding physician of the mechanism of the repression will enable him to guide the patient through a confession and mental review of the trouble, thus releasing the tension, readjusting the ideas and solving the problem.

A cultured man of excellent mind recently came to me the victim of a nervous breakdown. Seaching physical and laboratory examination brought out nothing. Psychoanalysis, however, revealed a life of sexual repression and conflict. The patient had remained continent till his marriage at the age of 28, and had never masturbated. Since marriage he had taken little interest in the sexual part of the relationship. On the other hand, he had developed an inordinate interest in and affection for boys. At the time of the examination he was educating five young men at his own expense, though not rich, and had gone so far as to legally adopt one young man. Here we see clearly a homesexual outburst of the repressed sexuality; or libido, of this patient. Such was the religious and ethical character of the sufferer, however, that he had absolutely repressed any tendency toward physical sexual relations with any of the young men in whom he was interested. Indeed, such a thought had not entered his consciousness, and he was at first most indignant when I explained the nature of his case. Nevertheless, a thorough understanding of the case, on his part, has greatly relieved him; he has lost his abnormal interest in his several proteges; has regained his nervous and emotional equalibrium; and has gone back to work.

To the next question: How is psychoanalysis accomplished in actual practice? Limited space does not permit of anything like a full answer.

The methods are three:

(1). Free association,

(2). Word association,

(3). Dream analysis.

The free association method of reaching ideas that have been crowded out of consciousness and into the subconscious mind consists simply of having the patient sit quietly and permit his thoughts to drift as they will. After a few minutes the physician has the patient to tell minutely just what passed through his mind and in what order. The patient must be cautioned not to control his ideas by volition, nor to consider any idea too delicate or absurd to report to the physician.

The word association test is made by reading to the patient, one at a time, say, a hundred words, and asking him to let his mental faculties play freely upon each word and report immediately what idea is brought to mind in response to the test word. A stop-watch is used to time the reaction interval in each separate instance; and those reaction intervals which are relatively long indicate that the ideas involved are connected with repressed experiences. This lead, together with the character of the ideas reported by the patient, often gives the physician a valuable clue to the hidden conflicts in the patient's mind.

Finally, if the patient is required at each sitting to report a dream which he remembers, the physician may then have the patient ponder unrestrictedly over each scene, character, name and idea of the dream, reporting each time the trains of thought thus brought to the surface. Dreams are now believed by most psychopathologists to be expressions of repressed ideas, though disguised and symbolic in form. Of the several methods of reaching the subconscious regions of the mind, dream analysis unquestionably offers the best means, the surest avenue of approach. .

To psychoanalysis there are limitations and objections. It is limited in its successful application to disorders of psychic origin, free from organic genesis; it is limited to persons under fifty years of age; and to those sufficently clear mentally and sufficiently intelligent and willing to co-operate with the physician.

Objections are the time and patience involved—some cases requiring many months. Moreover, the novelty of the procedure may inspire suspicion and derision in the minds of persons unacquainted with the real bearings of the procedure.

It is reassuring to those of us, however, who believe in the potency of psychoanalysis in selected cases to know that such conservative and experienced men as Wm. A. White, Superintendent of the Government Asylum, at Washington, practice and advocate it, not to mention Ernest Jones of Toronto and Jellieff of New York, who has written and translated books on the subject. Sigmund Freud, of the University of Vienna, originated this epoch-making system of psychotheropeusis, and to him is due the credit of solving several of the knotty problems of functional diseases of the mind and nervous system.

603 Candler Building.

